# Foraging for selenium: a comparison between hyperaccumulator and non-accumulator plant species

**DOI:** 10.1038/s41598-023-37249-z

**Published:** 2023-06-30

**Authors:** Sofia Montanari, Mirko Salinitro, Andrea Simoni, Claudio Ciavatta, Annalisa Tassoni

**Affiliations:** 1 Department of Biological Geological and Environmental Sciences, University of Bologna, Via Irnerio 42, 40126 Bologna, Italy; 2 Department of Agricultural and Food Sciences, University of Bologna, Viale Giuseppe Fanin 40, 40127 Bologna, Italy; 3 Centro Interdipartimentale di Ricerca Industriale sull’Agroalimentare, University of Bologna, Via Quinto Bucci 336, 47521 Cesena, Italy

**Keywords:** Plant development, Plant ecology, Plant physiology, Tropism

## Abstract

Selenium (Se) hyperaccumulators are a unique group of plants that can accumulate this element in their aerial parts at concentrations exceeding 100 mg kgDW^−1^. These plants actively search for Se in the soil, a phenomenon known as *root foraging*, reported to date only by few studies. In this study, the effect of localized Se enrichment, in the form of selenite and selenate, was investigated on the root architecture of two Se-hyperaccumulators (*Stanleya pinnata* and *Astragalus bisulcatus*) and two non-accumulators (*Brassica juncea* and *Medicago sativa*). Rhizoboxes were divided into two halves: one half was filled with control soil while the other with selenate or selenite (30 mg kgDW^−1^) spiked soil. Seedling were transferred into the interface of the two soils and allowed to grow for three weeks under controlled light and temperature conditions. *Staneya pinnata* exhibited equal root density in both halves of the rhizobox when grown in control/control and selenite/control soil treatments. However, in the presence of selenate, *S. pinnata* developed 76% of the roots towards the selenate-enriched half, indicating an active root foraging. In contrast, *A. bisulcatus* and the non-accumulators *B. juncea* and *M. sativa* did not show any preferential distribution of roots. This study revealed that only *S. pinnata* showed the ability to detect and forage for Se when provided as selenate. Non-accumulators did not show any morphological or Se-accumulation difference associated with the presence of Se in soil in either form.

## Introduction

Selenium (Se) is a non-metallic element belonging to the chemical group 16 together with sulphur, with which shares a similar chemical behaviour. Its distribution widely varies among world’s regions due to different soil genesis and rock parent material. The average global Se content in soils ranges between 0.01 and 2.00 mg kgDW^−1^^1,2^, with the highest concentrations (up to 1500 mg kgDW^−1^) usually found in sedimentary rocks such organic-rich black shales and coals^3^. From the parent rocks, Se is redistributed in sediments, originating natural seleniferous soils (with > 5 mg kgDW^−1^ Se)^4–6^. On these substrates, Se-hyperaccumulator plants have evolved, and are defined as species able to uptake and store in their shoots Se in concentrations exceeding 100 mg kgDW^−1^ DW^7–9^.

Se-hyperaccumulation probably evolved to provide protection against herbivores and pathogens as well as allelopathic benefits against competing species^10^, and species having this capacity were first discovered in areas of the central Western United States^11^. Se-hyperaccumulation occurs in many species in particular across the Fabaceae, Brassicaceae and Asteraceae plant families^10^. Notably, the *Astragalus* genus (Fabaceae) has 25 different taxa capable of hyperaccumulating Se^12^. *Astragalus bisulcatus* (Hook.) A. Gray and *A. racemosus* Pursh., for example, have been reported to accumulate > 10,000 mg kgDW^−1^ DW of Se in shoots when growing on natural seleniferous soils^13^. *Stanleya pinnata* (Pursh) Britton is also a known hyperaccumulator belonging to the Brassicaceae family capable of accumulating up to 4000 mg kgDW^−1^ Se^12,14^. In addition to US native species, Se hyperaccumulators have also been documented on seleniferous soils of Queensland (Australia), where *Neptunia amplexicaulis* Domin. (Fabaceae) was reported to uptake up to 4000 mg kgDW^−1^ Se^15^.

Selenium in soils is present in four oxidation states: Se(-II), Se(0), Se(IV) and Se(VI)^3^. At natural redox conditions, selenate (Se(VI)) and selenite (Se(IV)) represent the predominant fractions of inorganic Se in soil, with the former being more soluble and available than the latter^16^. Selenite is dominant under acidic and reducing conditions, whereas, in alkaline and oxidizing conditions, selenate appears to be predominant^3,17^. Elemental selenium (Se(0)) and selenides (Se(-II)), tend to exist in reducing, acid and organic-rich conditions and they are mostly unavailable for plants^3^.

Important factors influencing Se bioavailability in soils are the presence of iron (Fe), aluminium (Al), manganese (Mn) oxides, and the quantity of organic matter and clay minerals. Iron, Al, and Mn oxides, having a large chelating ability, are able to adsorb the negatively charged Se(IV) and Se(VI)^18,19^. Organic matter tends to form organo-mineral associations that increase Se immobilization in soils^20^. Lastly, in acidic conditions, clay particles, can adsorb and immobilize Se^18^. As a result of all previously described effects, Se in soils is distributed in several fractions with different bioavailability^21^. These, starting from the most available, are respectively soluble-Se (F1), exchangeable Se (F2), oxide-bound Se (F3), and residual Se (F4)^18,22^. The last fraction includes Se associated to sulphides and silicate minerals, which is not available for plants^23^.

Selenate transport inside root cells occurs through sulphate transporters, whereas phosphate transporters and anion channels are involved in the uptake of selenite^24^. Once inside, both selenite and selenate are known to cause oxidative stress at high concentration^25^. To cope with this, Se-hyperaccumulators convert most of the inorganic Se to organic forms mainly represented, in plant tissues, by methyl-SeCys (> 80%)^26^, although other compounds can be detected depending on the species, such as selenocystathionine (SeC) in *Stanleya pinnata*^27^, and selenomethionine (SeMet) in *Neptunia amplexicaulis*^28^. Hyperaccumulators can modify their root architecture in response to nutrient deficiencies or to the presence of their target accumulated element in the soil^29,30^. This process called *root foraging*, involves roots proliferating towards microenvironments in the soil where resources are more abundant for plant^31^.

Several studies have confirmed the ability of the Zn-hyperaccumulator *Noccaea caerulescens* to forage for zinc (Zn), cadmium (Cd) and nickel (Ni)^29,32–34^, while few reports are available for Se. Recently, a localized root proliferation in the Se-hyperaccumulator *Neptunia amplexicaulis*^35^ was reported, while previous studies have showed evidence for Se root foraging in *S. pinnata* and *A. bisulcatus* Se-hyperaccumulator species^36^. However, to the best of our knowledge, no data were previously published regarding the differential root foraging on selenite or selenate and the comparison with non-accumulator species.

The present study used the rhizobox approach aiming at: (i) investigating the influence of localized Se enrichment on root architecture and foraging of two Se-hyperaccumulators (*S. pinnata* and *A. bisulcatus*) and two non-accumulators (*Brassica juncea* L. and *Medicago sativa* L.) belonging to the same botanical families of selected hyperaccumulators; (ii) comparing the two main selenium forms (selenite and selenate) in influencing root foraging and architecture in hyperaccumulators and non-accumulators; (iii) assessing Se soil fractioning (soluble, exchangeable, oxide-bound and residual) before and after plant cultivation.

The presented findings provide insights into the root foraging capacity of both Se-hyperaccumulators and non-accumulators and will have significant implications for the future development of Se decontamination strategies using hyperaccumulators (i.e. by using species with pronounced root foraging behavior) and for crop Se-biofortification.

## Materials and methods

### Rhizobox preparation

The growing medium was composed by 80% w/w fine sand (< 0.5 mm) and 20% w/w sieved acid peat (< 2.5 mm). Part of the soil was spiked with a solution of sodium selenate (Na_2_SeO_4_) or with sodium selenite (Na_2_SeO_3_), to obtain a final soil concentration of 30 mg kg^−1^ Se DW. The remaining soil was moistened with deionized water and used as control. Spiked and control soils were stored in closed plastic bags for two weeks to allow equilibration. Selenium salts were purchased from Merck (Darmstadt, Germany).

**Figure 1:**
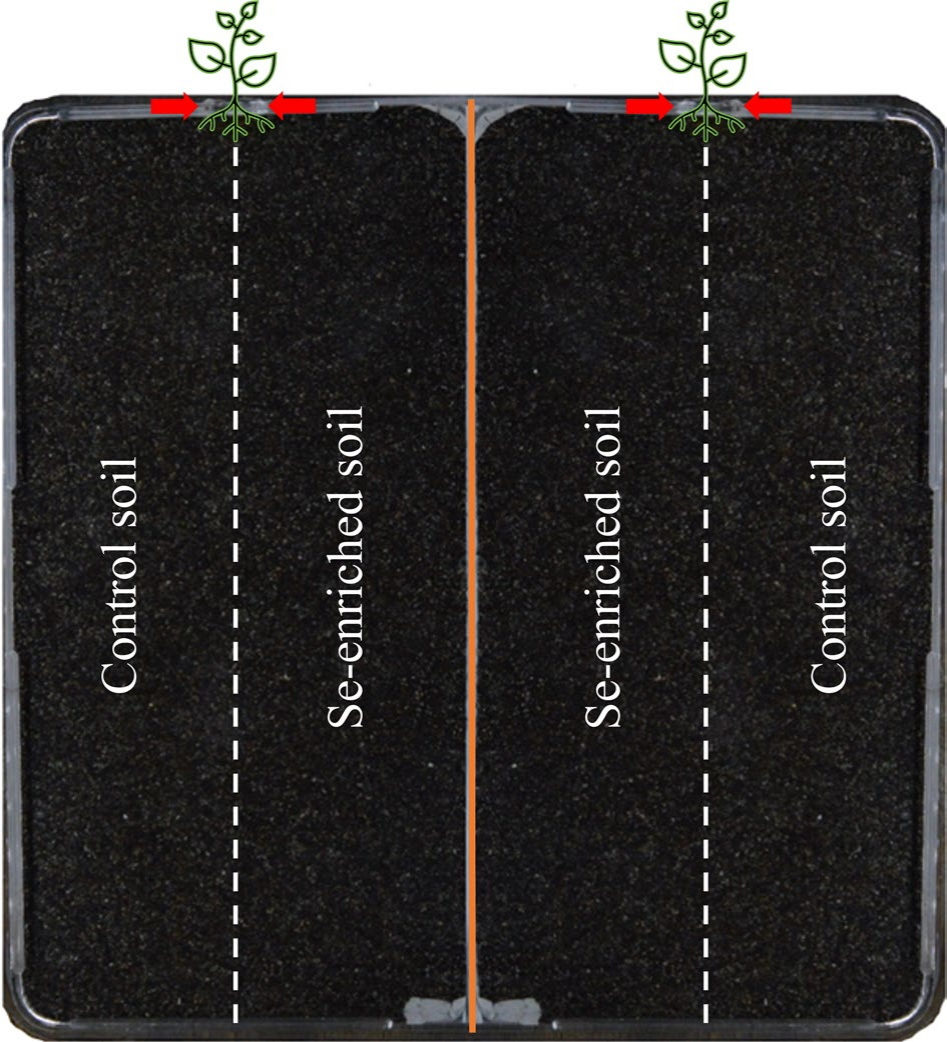
Example of a rhizobox setup. The orange line indicates the polystyrene physical barrier placed to separate the two replicates of each rhizobox. Dotted lines indicate the interface between the Se-enriched and control soils. Red arrows indicate the top holes where seedlings were inserted.

Forty-eight rhizoboxes were prepared using squared 23 × 23 cm Petri dishes, each rhizobox hosted two replicate plants and in the central part of the rhizobox a polystyrene septum was placed to physically separate the rhizosphere of the two replicate plant individuals (Fig. 1).

Rhizoboxes were filled with control/selenate, control/selenite or control/control soils. Eight replicate plants per treatment were cultivated and, to take into consideration root chirality, in each rhizobox the Se-enriched soil was always placed in the central part so that for one plant was on the right and for the other on the left (Fig. 1). A rigid plastic sheet was used to separately fill each half of the same rhizobox, then removed to allow the contact of the two soil types.

### Seedlings production and transfer

Selected Se-hyperaccumulators were *S. pinnata* (Pursh) (Brassicaceae) and *A. bisulcatus* (Hook.) A. Gray (Fabaceae) while non-accumulator plants *B. juncea* L. and *M. sativa* L. were selected as they belong to the same botanical family of the hyperaccumulators. Seeds of the Se-hyperaccumulators were purchased from Prairie Moon Nursery (Winona, Minnesota, USA) while those of non-accumulators were obtained from the Department of Agricultural and Food Sciences (University of Bologna, Bologna, Italy). Before germination, *A. bisulcatus* seeds were scarified using sandpaper. All seeds were hydrated with tap water for 4 h, then placed on moist paper for germination in the dark at 25 °C. The germination occurred in 5–7 days, then the seedlings were transferred in the rhizoboxes at cotyledon stage and watered with 1 ml of deionised water to assure establishment. Rhizoboxes were not watered anymore to avoid Se leaching or delocalization between the two halves and were covered with aluminium foil to avoid the light penetration in the soil. Then they were arranged with an angle of 45° to force roots growing on the Petri surface for observation. All plants were cultivated for 3 weeks in a growth chamber with 16/8 h light/dark at 25 °C and 60–80% humidity. A photosynthetic photon flux density (PPFD) of 650 μmol m^−2^ s^−1^ was provided with LED lights (TLED 42W full spectrum, Secret Jardin, Belgium)**.**

### Sample collection and analysis

For each treatment, five g of soil from each rhizobox half were collected after the equilibration period (2 weeks) (initial soil) and at the end of the 3-weeks cultivation period (post-cultivation soil). Soil samples were dried in a ventilated oven at 40 °C for 48 h and stored in plastic bags until analysis. Plant shoots and roots were separately collected. Roots from the two halves of the rhizobox could not be collected separately but were pulled together to achieve a total biomass suitable for further analysis. Plant parts were dried and stored similarly to soil.

Aqua regia digestion was performed on soil samples to quantify total elements, following a modified method according to Hseu et al.^37^. 1.5 g DW of soil was weighted and placed in Teflon digestion tubes for close vessel digestion together with 6 ml of 37% v/v HCl, 2 ml of 69% v/v HNO_3_ and 0.5 ml of 35% v/v H_2_O_2_. All the reagents at trace element analysis grade, were purchased from Merck (Darmstadt, Germany). Digestions were performed using a STARD D microwave system (Milestone, Sorisole, BG, Italy) with the following cycle: 2 min at 250 Watt, 2 min at 400 Watt, 1 min at 0 Watt and 3 min at 750 Watt. After digestion, all samples were brought to a volume of 20 ml with milliQ water.

For plant sample digestions, 0.1 g of dry weight (g DW) of roots/shoots were weighted and placed in Teflon digestion tubes with 69% v/v HNO_3_ and 0.5 ml of 35% v/v H_2_O_2_ following a modified method from Tüzen, (2003)^38^. Tubes were subsequently placed in the microwave and undergone the following digestion cycle: 2 min at 250 Watt, 2 min at 400 Watt, 1 min at 0 Watt and 2 min at 600 Watt. After digestion, samples were brought up to the volume of 10 ml. All samples were filtered with Whatman 42 ashless filter paper (Maidstone, UK).

The quantification of trace elements was carried out with a Spectro Arcos II ICP-OES (Ametek, Berwyn, Pennsylvania, US). The limits of quantification of the analysed elements were 0.067 mg kg^−1^, 0.037 mg kg^−1^, 0.042 mg kg^−1^, 0.0054 mg kg^−1^, 0.0065 mg kg^−1^, 0.00031 mg kg^−1^, 0.0017 mg kg^−1^, 0.0113 mg kg^−1^ and 0.00038 mg kg^−1^ for Ca, K, Mg, Na, P, S, Fe, Se, and Zn, respectively. For quality control, BCR-143R sewage sludge amended soil (JRC-Joint Research Centre, Geel, Belgium) SRM 1570a spinach leaves (NIST, Maryland, US) were digested and analysed together with soil and plant samples. Recovery rates of certified elements were > 95%. Quality control solutions were also included during measurements to assure instrumental stability. Data were expressed as mg of element per kg sample dry weight (mg kgDW^-1^).

### Selenium sequential extraction

Selenium sequential extraction was performed following a modified method from Chao et al.^22^. The method quantified four Se fractions (F) showing different bioavailability: soluble Se (F1), clay and organic matter-bound (or exchangeable) Se (F2), Fe-Al-Mn oxide-bound Se (F3), residual Se (F4). To extract F1, 10 gDW of soil were weighted and placed in 50 ml tubes with 25 ml of 0.25 M KCl, samples were shaken for 30 min at room temperature. Tubes were subsequently centrifuged for 20 min at 4,000 rpm. The supernatant was filtered with 0.45 μm Acrodisc syringe filter (Pall Corporation, Washington, USA). To extract F2, the residual soil from F1 was mixed with 25 ml of 0.1 M KH_2_PO_4_ then shaken, centrifuged and filtered as before. To bring into solution F3, residual soil from F2 was mixed with 25 ml of 4 M HCl. Samples were incubated for 45 min at 95 °C then, centrifuged and filtered as above. F3 residual soil was oven-dried at 40 °C for 2 days and to quantify residual Se (F4), 1.5 g of each dried sample was weighted and analysed by ICP-OES as previously described.

### Image processing

After three weeks of cultivation rhizoboxes were photographed with a Nikon D3200 camera (Chiyoda, Tokyo, Japan). All images were first modified using the software GIMP 2.10.32 (https://www.gimp.org/), to homogenize the background soil, and then transformed into binary (only black and white pixels) using the *Binary* function of the software ImageJ 1.8.0 (https://imagej.nih.gov/ij/). Roots density (%) in each half of the rhizoboxes was calculated as the ratio of white pixel number / number of total pixel of each analysed half.

### Data analysis

All variables were tested for homogeneity using Levene’s test for homogeneity of variance and for normality using Shapiro–Wilk normality test from the package *car* (https://cran.r-project.org/web/packages/car/index.html). When data resulted parametric, analysis of variance (ANOVA) followed by Tukey HSD post-hoc test were used to evaluate differences among compared groups. When data resulted non-parametric, the analysis was performed using Kruskal–Wallis Rank Sum test followed by Dunn's test for multiple comparisons using Rank Sums post-hoc test (https://cran.rproject.org/web/packages/dunn.test/index.html). Student’s T-test and Wilcoxon Rank Sum tests were used to compare roots density in rhizobox halves. Statistical and graphical analysis were carried out using the software R 4.0.2 and Microsoft Excel^©^ for Microsoft 365.

### Research involving plants

The present research complies with relevant institutional, national, and international guidelines and legislation (IUCN, CITES). The used species are not protected by any specific laws and were cultivated within the University of Bologna starting from purchased or self-produced germplasm. Nothing was collected from the wild. The formal identification of the used species was carried out by M. Salinitro in collaboration with experts of the Botanical Garden of Bologna’s University. Voucher specimens of the selected species were collected and deposited at the Herbarium of the University of Bologna.

## Results

### Root architecture in response to localized selenium

**Figure 2:**
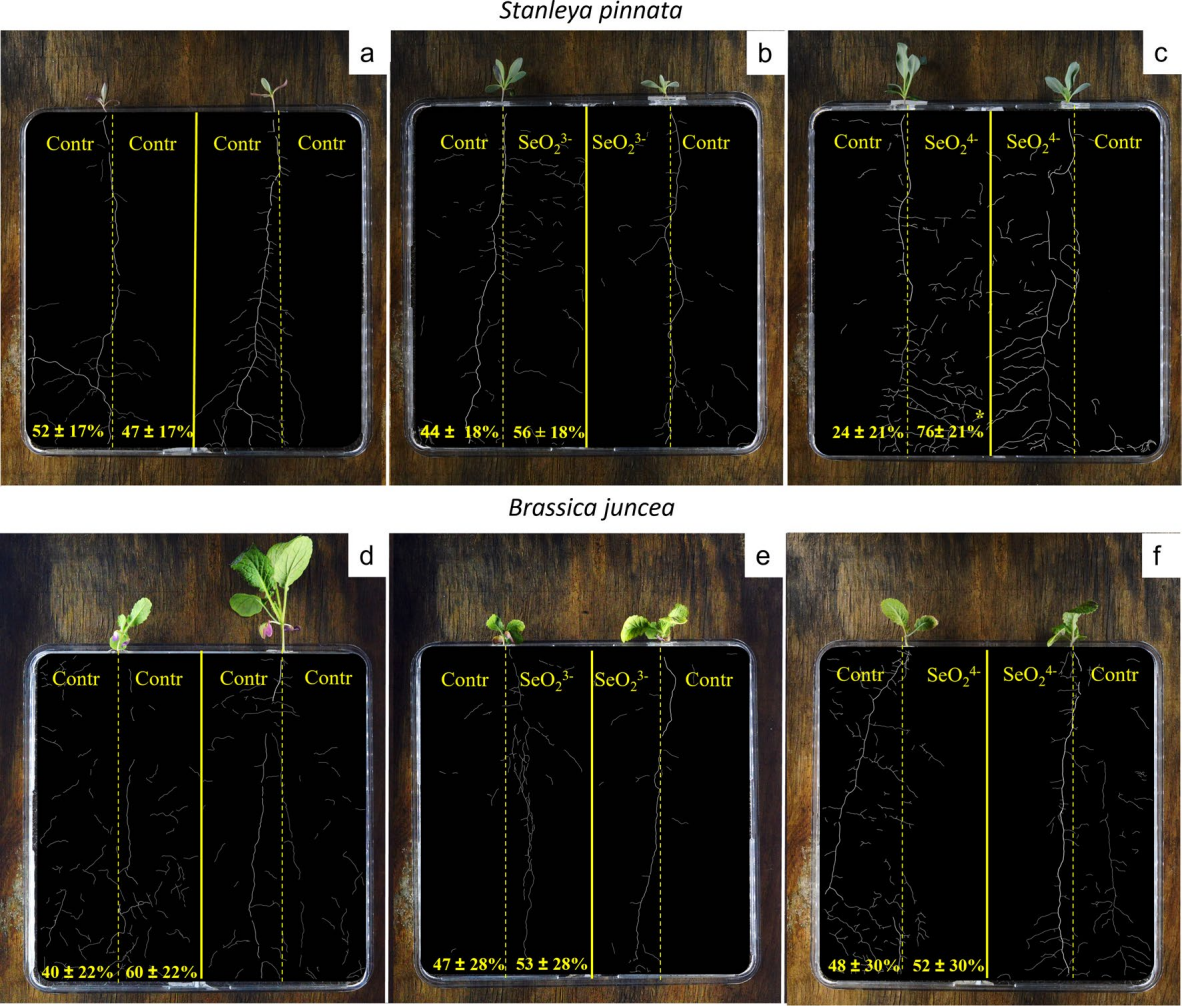
Root density in Se-hyperaccumulator *S. pinnata* and non-accumulator *B. juncea* (both of Brassicaceae family). (**a,d**) control/control treatment; (**b,e**) control/selenite (30 mg kgDW^−1^) treatment; (**c,f**) control/selenate (30 mg kgDW^−1^) treatment. Reported percentages are the average of 8 biological replicates for each treatment (n = 8). The star symbol (*) indicates a significant difference in root density among the two halves after Student’s/Wilcoxon Rank Sum Tests (*p* < 0.05) (n = 8).

When grown in control/control and selenite/control treatments, the Se-hyperaccumulator *S. pinnata* exhibited equal root density in both halves of the rhizobox (*p* = 0.53*, p* = 0.21) (Fig. 2a,b). However, in the presence of selenate, *S. pinnata* developed 76% of its roots towards the selenate-enriched half, and 24% in the control half (*p* < 0.05) (Fig. 2c). In contrast, *B. juncea,* the related non-accumulator of the same botanical family, did not show any preferential root distribution in either selenate (*p* = 0.78) or selenite (*p* = 0.98) treatments (Fig. 2d–f).

**Figure 3:**
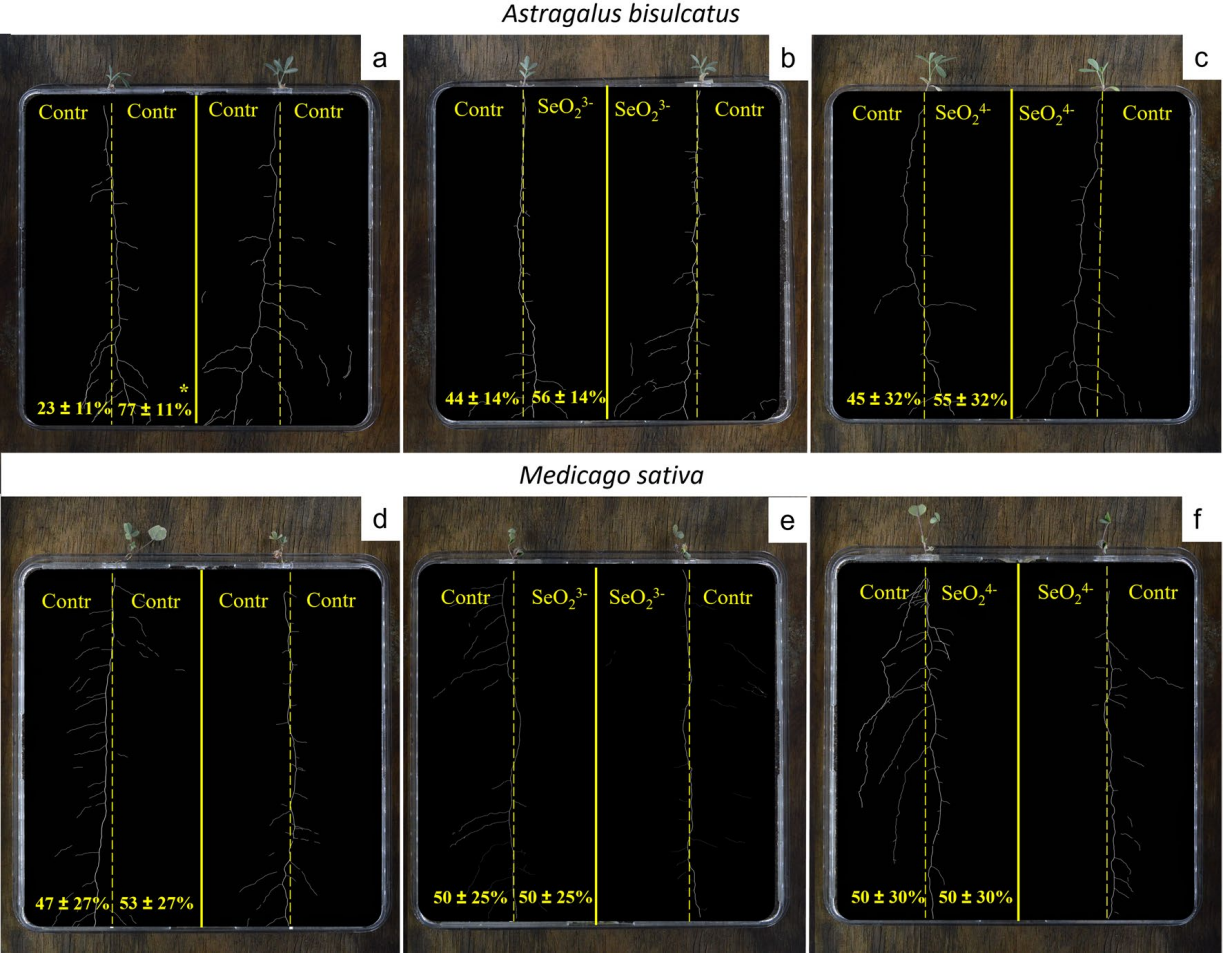
Root density in *A. bisulcatus* and *M. sativa* (both of Fabaceae family). (**a,d**) control/control treatment; (**b,e**) control/selenite (30 mg kgDW^−1^) treatment; (**c,f**) control/selenate (30 mg kgDW^−1^) treatment. Reported percentages are the average of 8 biological replicates for each treatment (n = 8). Root density data were not significantly different among the two halves after Student’s/Wilcoxon Rank Sum Tests (*p* < 0.05) (n = 8).

*A. bisulcatus* Se-hyperaccumulator did not exhibit any sign of root foraging in selenite or selenate treatments, developing equal root density in both Se-enriched and control halves (*p* = 0.10*, p* = 0.61) (Fig. 3b,c). In control/control rhizoboxes (Fig. 3a), *A. bisulcatus* roots were unevenly distributed in the two halves (77% and 13%, respectively, *p* < 0.05). Similarly to *B. juncea*, the non-accumulator *M. sativa* (of the same botanical family of *A. bisulcatus*), did not show any preferential root distribution in any treatment and was not sensitive to the presence of either selenite (*p* = 0.96) or selenate (*p* = 0.97) (Fig. 3d–f). In a few cases, both *B. juncea* and *M. sativa* showed signs of Se-avoidance, developing more roots in the control part than in the Se-enriched part, although these results were not statistically significant.

### Soil elemental analysis

The total element concentration in soil is shown in Table 1. The average concentration of the analysed macronutrients before plant growth were 31,900 mg kgDW^−1^ Ca, 1300 mg kgDW^−1^ K, 2200 mg kgDW^−1^ Mg, 200 mg kgDW^−1^ Na, 160 mg kgDW^−1^ P and 550 mg kgDW^−1^ S. Overall, no significant differences were recorded after plant cultivation, except for a 4.8% decrease in the concentration of Ca in selenate/control rhizobox with *M. sativa* (*p* < 0.05, Table 1). The initial concentrations of the Fe and Zn micronutrients was found to be on average 6700 and 25 mg kgDW^-1^, respectively. Zinc decreased of the 23% after *M. sativa* cultivation only in control/control rhizoboxes (*p* < 0.05).

**Table 1:** Macronutrients, micronutrients and selenium total concentrations in initial soil and post-cultivation soil.

	Species	Treatment	Ca (mg kgDW^−1^)	K (mg kgDW^−1^)	Mg (mg kgDW^−1^)	Na (mg kgDW^−1^)	P (mg kgDW^−1^)	S (mg kgDW^−1^)	Fe (mg kgDW^−1^)	Zn (mg kgDW^−1^)	Se (mg kgDW^−1^)
Initial soil	**–**	Control	32,000 ± 700^A^	1300 ± 100^A^	2220 ± 90^A^	220 ± 10^x^	160 ± 20^A^	630 ± 40^A^	6600 ± 200^A^	26 ± 2^A^	< LoD
		Selenite	32,000 ± 1,000^a^	1330 ± 30^a^	2300 ± 100^a^	204 ± 7^A^	180 ± 10^a^	550 ± 50^a^	6800 ± 200^a^	28 ± 3^a^	22 ± 1^a^
		Selenate	33,000 ± 1,000^w^	1370 ± 90^w^	2230 ± 60^w^	220 ± 20^**a**^	171 ± 7^w^	590 ± 40^w^	6900 ± 200^w^	24 ± 1^w^	23 ± 3^w^
Post-cultivation soil	*S. pinnata*	Control	31,900 ± 200^A^	1400 ± 100^B^	2200 ± 100^A^	190 ± 10^y^	160 ± 10^A^	580 ± 50^A^	6700 ± 300^A^	26 ± 1^A^	< LoD
		Selenite	31,700 ± 600^a^	1400 ± 200^a^	2400 ± 200^a^	230 ± 60^A^	180 ± 50^a^	510 ± 70^a^	6800 ± 300^a^	27 ± 5^a^	25 ± 3^a^
		Selenate	32,300 ± 200^w^	1400 ± 200^w^	2400 ± 400^w^	190 ± 20^**a**^	200 ± 80^w^	490 ± 60^w^	6700 ± 400^w^	24 ± 3^w^	25 ± 3^w^
	*B. juncea*	Control	31,500 ± 700^A^	1500 ± 300^A^	2400 ± 300^A^	220 ± 10^x^	220 ± 90^A^	540 ± 60^A^	6600 ± 70^A^	29 ± 4^A^	< LoD
		Selenite	32,300 ± 600^a^	1400 ± 100^a^	2300 ± 200^a^	210 ± 30^A^	170 ± 50^a^	480 ± 100^a^	7200 ± 400^b^	25 ± 3^a^	24 ± 2^a^
		Selenate	32,000 ± 400^w^	1500 ± 200^w^	2200 ± 200^w^	220 ± 30^**a**^	170 ± 30^w^	500 ± 50^w^	7300 ± 100^x^	26 ± 2^w^	27 ± 2^w^
	*A. bisulcatus*	Control	32,000 ± 300^A^	1100 ± 100^A^	2200 ± 100^A^	190 ± 40^y^	140 ± 20^A^	670 ± 90^A^	6600 ± 200^A^	24 ± 1^A^	< LoD
		Selenite	32,000 ± 500^a^	1300 ± 100^a^	2200 ± 100^a^	190 ± 30^A^	160 ± 10^a^	530 ± 60^a^	6620 ± 70^a^	24 ± 1^a^	20 ± 1^b^
		Selenate	31,900 ± 200^w^	1300 ± 100^w^	2000 ± 200^w^	200 ± 30^**a**^	150 ± 20^w^	600 ± 100^w^	6,600 ± 300^w^	23 ± 1^w^	20 ± 2^x^
	*M. sativa*	Control	31,800 ± 200^A^	1150 ± 70^A^	2100 ± 200^A^	173 ± 9^y^	120 ± 20^A^	630 ± 50^A^	6300 ± 200^A^	20 ± 1^B^	< LoD
		Selenite	31,500 ± 300^a^	1300 ± 200^a^	2200 ± 100^a^	180 ± 20^A^	150 ± 20^a^	500 ± 100^a^	6700 ± 200^a^	24 ± 1^a^	20 ± 1^b^
		Selenate	31,400 ± 500^x^	1300 ± 200^w^	2100 ± 100^w^	200 ± 30^**a**^	160 ± 30^w^	510 ± 70^w^	6700 ± 100^w^	25 ± 2^w^	21 ± 2^x^

Values are expressed in mg kgDW^−1^.

*LoD* Limit of detection.

Letters indicate statistically significant difference for a given element before/after cultivation among different species, after ANOVA/Kruskal–Wallis tests, followed by Tukey HSD/Dunn’s tests, (*p* < 0.05). Upper-case (A–D) letters refer to control soil; lower-case (a–d) letters refer to selenite soils; upper-case (x–z) letters refers to selenate soils (n = 5).

The average initial concentrations of Se were 22 and 23 mg kgDW^−1^ for selenite and selenate-enriched soils, respectively. A decrease in Se after *A. bisulcatus* and *M. sativa* cultivation was recorded both in selenite and selenate-enriched soils (average of 20 mg kgDW^−1^, *p* < 0.05). An slight increase (+ 10%) in total Se was detected after the cultivation of *S. pinnata* but this was probably caused by an analytical error.

### Selenium fractioning in soil

**Figure 4:**
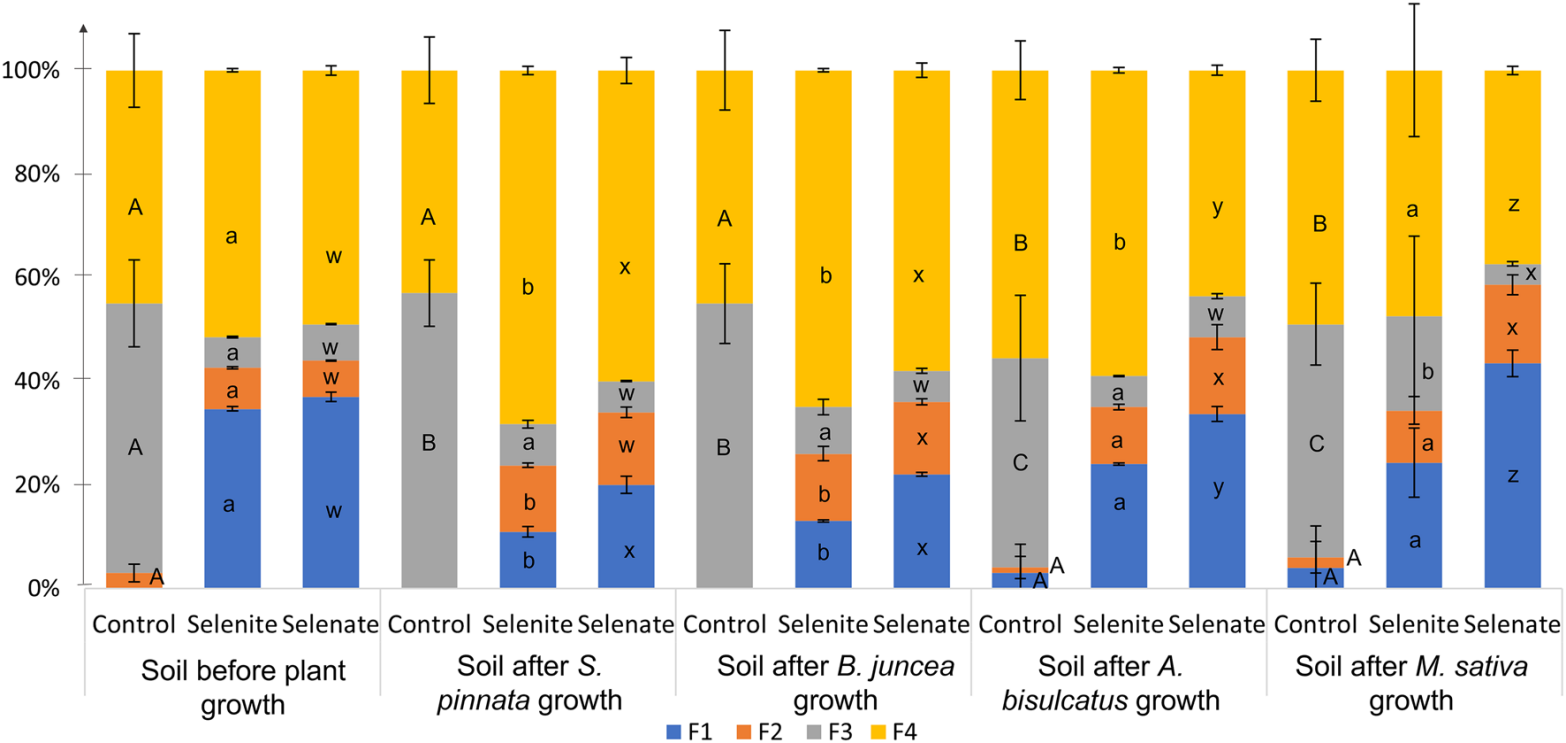
Selenium fractioning in the soils before (initial soil) and after cultivation (post-cultivation soil) of the four plant species. Blue: soluble fraction (F1); orange: exchangeable fraction (F2); gray: oxide-bound fraction (F3); yellow: residual fraction (F4). Different letters indicate statistically significant differences among fractions within the soil before and after cultivation of the same plant species (ANOVA/Kruskal–Wallis tests, followed by Tukey HSD/Dunn’s tests, *p* < 0.05). Upper-case (A–D) letters refer to control soils; lower-case (a-d) letters refer to selenite soils; lower-case (x–z) letters refer to selenate soils (n = 4).

Selenium fractioning was evaluated in the soils before and after plant growth (Fig. 4). Before plant cultivation, Se soluble fraction (F1) in the selenite and selenate-enriched soils accounted for the 35% and 37% respectively, the exchangeable Se (F2) was 8% and 7%, the oxide-bound Se (F3) accounted for 6% and 7%, while the residual fraction (F4) was the most abundant one (52% and 49%, in selenite and selenate-enriched soil, respectively) (Fig. 4). Selenium was below the limit of detection (LoD) in control soils.

Overall, after plant cultivation the available fraction (F1) decreased of the 10–20% in all treatments. In particular in the hyperaccumulator *S. pinnata,* F1 passed from 36 to 15%, while F2 increased from 7.5% to 13.5% (average of selenate and selenite). F3 did not change after plant cultivation (*p* = 0.62), while F4 was significantly increased, passing from 50 to 65% (*p* < 0.05). In *A. bisulcatus* the depletion on F1 was less marked and accounted for the 11% for selenite and 3% in selenate treatments, while similar trends were detected other fractions. After the cultivation of *B. juncea*, selenium fractioning was similar to that reported for *S. pinnata*, conversely after the cultivation *M. sativa*, the depletion of F1 was not detected.

### Plant growth performances

**Figure 5:**
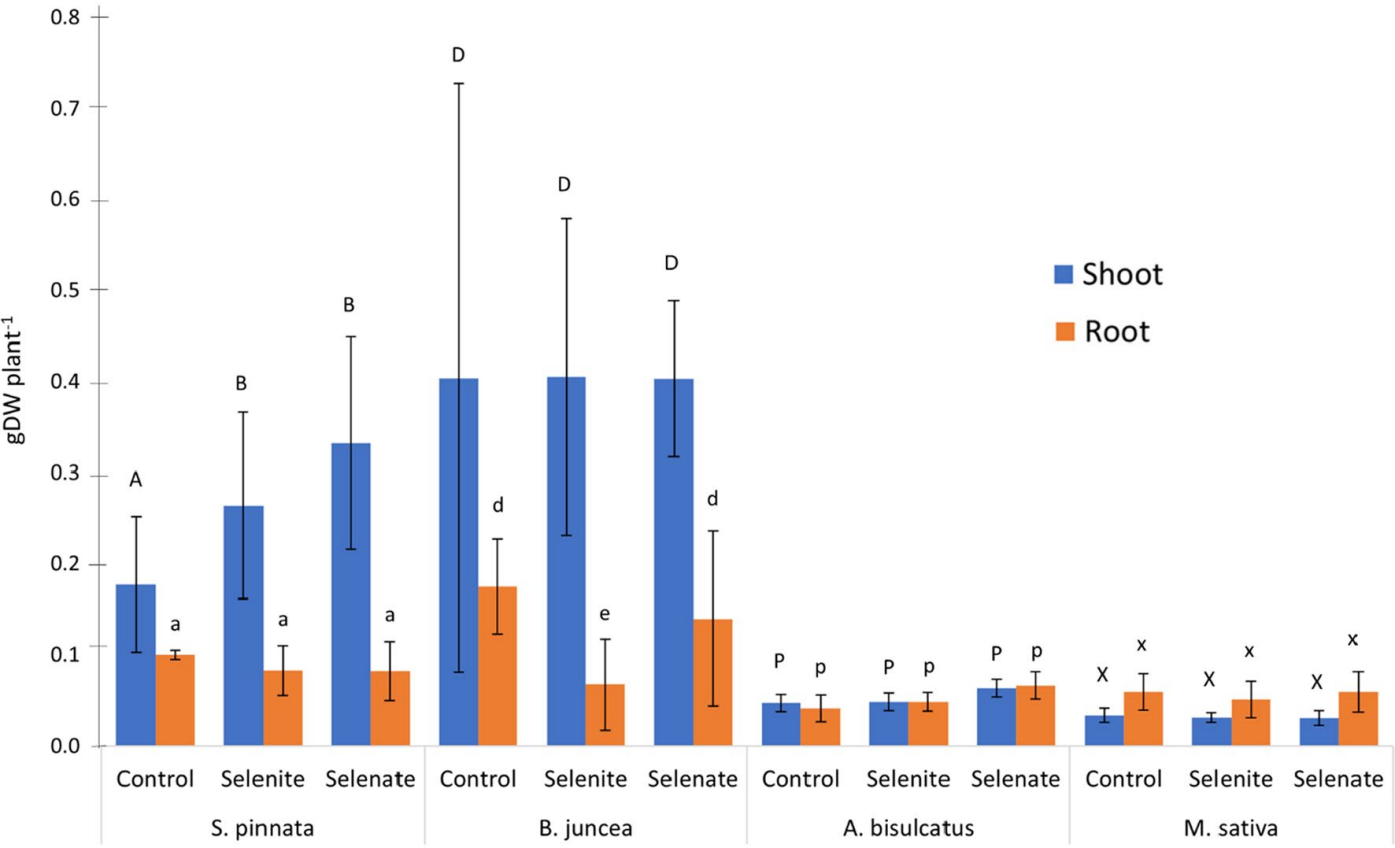
Shoot and root average biomass (gDW plant^−1^) in the different treatments. Different letters indicate statistically significant differences among treatments within the same plant species after ANOVA/Kruskal–Wallis tests, followed by Tukey HSD/Dunn’s tests, (*p* < 0.05). Upper-case letters refer to shoots, lower-case letters refer to roots. A–C and a–c: *S. pinnata*, D–F and d–f: *B. juncea*, P–T and p–t: *A. bisulcatus*, X–Z and x–z: *M. sativa,* (n = 4).

Figure 5 represents the average shoot and root dry weight of the plant species measured at the end of the cultivation period in the three different treatments (control/control, selenate/control, selenite/control). *S. pinnata* and *B. juncea* exhibited greater growth with an average harvestable shoot biomass among the three treatments of 0.24 and 0.36 gDW plant^−1^ respectively, compared to *A. bisulcatus* and *M. sativa*, with a shoot dry biomass of only 0.05 and 0.03 gDW plant^−1^ respectively, on average in the three treatments. A similar trend was observed for root biomass (Fig. 5). *S. pinnata* showed significantly higher shoot biomass in the rhizoboxes containing Se-enriched soils (0.31 gDW plant^−1^) compared to those containig control soil (0.19 gDW plant^−1^) (*p* < 0.05). No significant differences were recorded between selenite and selenate samples. Root biomass was similar in all treatments. In contrast, *B. juncea* shoot biomass did not exhibit significant differences among the three treatments (*p* = 0.72), although some individual plants grew more in the control rhizoboxes (0.40 gDW plant^−1^) compared to selenite and selenate ones (0.31 and 0.33 gDW plant^−1^ respectively). The root biomass showed a similar trend, with higher values in the control (0.17 gDW plant^−1^) compared to selenite and selenate (0.06 and 0.13 gDW plant^−1^) (*p* < 0.05). No significant differences were observed between shoot and root biomass in *A. bisulcatus* and *M. sativa* (*p* = 0.65).

### Selenium and sulphur accumulation

Selenium shoot concentration was found to be significantly higher in *S. pinnata* and *A. bisulcatus* compared to non-accumulators (*p* < 0.05) (Table 2). Higher concentrations of this element were observed in selenate treatments (1950 and 2400 mg kgDW^−1^) compared to selenite ones (1320 and 530 mg kgDW^−1^,) in *S. pinnata* and *A. bisulcatus* shoots respectively. A similar trend was observed for shoot and root Se concentration of non-accumulator species (Table 2). Sulphur was also measured since its accumulation is closely linked to that of Se and differs in hyperaccumulator and non-accumulator plants. Overall, shoot and root S concentrations were higher in the plant samples from selenate rhizoboxes than in those from selenite ones (*p* < 0.05). Among the four species, *S. pinnata* exhibited the highest S concentrations in both shoots and roots (26,000 and 12,000 mg kgDW^−1^, respectively, average all treatments) whereas *M. sativa* accumulated lower concentrations of this element (average of 4500 and 4300 mg kgDW^−1^, respectively). In selenate/control treatments, significantly higher Se/S ratios were obtained for *S. pinnata* and *A. bisulcatus* shoots (0.09 and 0.10, respectively) and roots (0.061 and 0.068, respectively) compared to the non-accumulator species. Lastly, Se/S ratios obtained in selenite-enriched soils did not show significant differences between hyperaccumulators and non-accumulator species.

**Table 2:** Content of Se and S and their ratio in shoot and roots of the different plant species.

Species	Treatment	Shoot concentration (mg kgDW^−1^)		Se/S	Root concentration (mg kgDW^−1^)		Se/S
		Se	S		Se	S	
*S. pinnata*	Selenite	1320 ± 40^a^	28,000 ± 700^a^	0.05 ± 0.02	340 ± 10^a^	12,000 ± 300^a^	0.029 ± 0.001
	Selenate	1950 ± 70^b^	23,000 ± 4000^a^	0.09 ± 0.02	710 ± 20^b^	11,800 ± 400^a^	0.061 ± 0.001
*B. juncea*	Selenite	250 ± 20^a^	10,000 ± 5000^a^	0.03 ± 0.01	173 ± 3^a^	6200 ± 15^a^	0.0279 ± 0.0004
	Selenate	480 ± 10^b^	29,000 ± 2000^b^	0.016 ± 0.001	299 ± 9^b^	6500 ± 200^b^	0.046 ± 0.002
*A. bisulcatus*	Selenite	530 ± 70^a^	13,000 ± 1000^a^	0.041 ± 0.001	128 ± 2^a^	6000 ± 200^a^	0.0212 ± 0.0002
	Selenate	2400 ± 200^b^	24,000 ± 5000 ^b^	0.098 ± 0.008	500 ± 20^b^	7500 ± 200^b^	0.068 ± 0.001
*M. sativa*	Selenite	150 ± 20^a^	4000 ± 1000^a^	0.04 ± 0.01	113 ± 1^a^	4500 ± 100^a^	0.025 ± 0.001
	Selenate	300 ± 80^b^	5000 ± 1000^a^	0.061 ± 0.001	201 ± 3^b^	4200 ± 100^b^	0.048 ± 0.001

Different letters indicate statistically significant differences among selenate and selenite treatments in each species and plant organ after ANOVA/Kruskal–Wallis tests, followed by Tukey HSD/Dunn’s tests, (*p* < 0.05) (n = 8).

## Discussion

Only *S. pinnata* plants cultivated in the selenate/control rhizoboxes showed significant evidence of root foraging, producing most of the roots (75%) in the selenate-enriched soil (Fig. 2). This result was in line with previous results^36^ which reported root foraging in *S. pinnata* when cultivated for two months in pots. Conversely, *A. bisulcatus* (Fig. 3), did not show any preferential distribution of roots towards the Se-enriched part (either selenate or selenite) of the rhizobox as also observed by Goodson et al.^36^, who reported a main vertical development of roots at the expenses of lateral branching. Other Se-hyperaccumulators have been reported to forage Se, including *Symphyotrichum ericoides* (L.) G.L.Nesom which showed a preferential root development towards Se, in terms of biomass, length and width^39^. Recently, also *Neptunia amplexicaulis*, a Se-hyperaccumulator native of Queensland (Australia), was reported to forage Se when this element was in the soil in soluble form^35^.

The present study also reported that some individuals of non-accumulators *B. juncea* and *M. sativa* showed signs of avoidance, having a lateral root proliferation shifted towards the control soil (not statistically significant).This type of behaviour in *B. juncea* was never reported in previous studies which analysed root architecture of this species under similar conditions^36^. On the other hand, *Medicago sativa*, the second non-accumulator plant here used, was never tested before for Se foraging.

Plant growth did not have a significant impact on soil macro- and micro-nutrients because of the short period of plant cultivation (Table 1). Nevertheless, the plant growth impacted on Se content and fractioning. The Se concentration in soil before cultivation resulted considerably lower than expected 22 and 23 mg kgDW^-1^ instead of 30 mg kgDW^-1^ for both selenite and selenate initially provided during soil preparation. This could have been caused by Se volatilization probably occurring during the equilibration period (e.g. methylation by soil microorganisms)^1,20^ or during soil processing prior to analysis (e.g. drying and mineralization).

Selenium distribution in the different fractions differed between selenite and selenate enriched soil (Fig. 4). After plant cultivation in the selenite soils, the residual fraction (F4) accounted for the 69% of the total, compared to the 48% of selenate soils. Soluble and exchangeable Se forms (F1-F2) were in fact preferentially removed by plant roots, triggering Se release from less mobile fractions (i.e. F4)^18^. In line with this, soluble Se (F1) was higher in soils spiked with selenate (29%, on average) than in those with selenite (14%, on average). Selenate is in fact known to be less toxic and more absorbed by plant roots with respect to selenite^10,18^. Since soluble Se represents the most available form for plants^18,40^, fraction F1 significantly decreased (about − 25%) after *S. pinnata* cultivation, especially in the soils spiked with selenate. Plants can occasionally extract Se from F3-F4 fractions, for examples when roots come in direct contact with this pool^18^. However due to restricted root development this effect was not observed in the present experiment. Overall, Se distribution in residual fraction (F4) and oxide-bound fraction (F3) resulted in line with the values found soil amended with 0.5 mg kgDW^−1^ selenite after rice (*Oryza sativa* L.) cultivation^41^ (60–80% of Se in F4 and 4–10% in the F3). As further confirmation, plant biomasses were larger in selenate/control rhizoboxes compared to selenite ones for all the tested species including non-accumulators (Fig. 5). Similar results were obtained for lettuce (*Lactuca sativa* L.) and cucumber (*Cucumis sativus* L.) whose biomass decreased of 60% more in plants exposed to selenite compared to those subjected to the same selenate concentration^42,43^.

As expected hyperaccumulators, especially *S. pinnata*, produced a larger shoot biomass (+23%) in presence of Se, whereas non-accumulator plants showed the opposite trend (Fig. 5). Previous studies confirmed that Se represents a beneficial element for hyperaccumulators stimulating their growth^10,44^.

Selenium accumulation in *S. pinnata* and *A. bisulcatus* was higher compared to the non-accumulators (Table 2) and both species accumulated more Se when cultivated on selenate-enriched soils compared to the selenite-enriched ones. The Se concentrations reached in shoots of these plants (1950 and 2400 mg kgDW^−1^, respectively) after only 3 weeks of cultivation on 30 mg/kg DW Se spiked soil, resulted comparable to those detected in the same plants grown under natural conditions^45,46^.

Interestingly, when cultivated on selenate-enriched soil, also *B. juncea* accumulated notable quantities of Se (476 mg kg DW^−1^ in shoots) (Table 2). This result was in line with previous studies carried out on the same species, with an accumulation of about 500 mg kgDW^−1^ Se when grown for 4 weeks on soil treated with 20 µM selenate^47^. Due to its ability to store significant quantities of Se in the shoot biomass, *B. juncea* has been sometimes reported as a secondary Se accumulator^27^. Lastly, *M. sativa* resulted the least efficient species in Se accumulation with 297 mg kgDW^−1^ Se in shoots after growth on soil spiked with selenate (Table 2). Similarly, a previous study demonstrated that this species grown for longer period (6 months) on soil spiked with 20 mg kgDW^−1^ selenate never overcame 600 mg kgDW^−1^ Se in its shoots^48^. The present results showed that in selenate treatment the Se/S ratio was 30–40% higher in hyperaccumulator than in non-accumulator plant species. It has in fact been demonstrated that Se-hyperaccumulators, although using the same strategy than non-accumulators in taking up Se, seem to be able to distinguish between Se and S^27^. This capacity results in much higher Se/S ratio in their tissues compared to non-accumulator species^49^.

## Conclusions

The present study provides new insights on the ability of the Se-hyperaccumulators *S. pinnata* and *A. bisulcatus* to and the non-accumulators *B. juncea* and *M. sativa* to forage for Se, and may have important implications for the future development of strategies aimed at managing Se in contaminated soils or for crop biofortification. Our results suggest that Se has a growth promoting effect on the hyperaccumulator *S. pinnata*, especially if provided in the form of selenate. *S. pinnata* proved to actively forage for selenate under the tested conditions, whereas this phenomenon was not observed when the plant was cultivated with selenite. *A. bisulcatus*, did not forage for Se as its root development was not affected by the presence of Se in both forms. At the tested concentrations, non-accumulators did not show any significant sign of Se toxicity in response to Se addition in either form. However, *B. juncea* and *M. sativa* showed occasionally signs of Se avoidance when cultivated in rhizoboxes with selenate, with roots developing preferentially in the control half.

Our results showed that selenate is more easily detected and preferentially taken up by plant roots compared to selenite and that the soluble and exchangeable fractions (F1, F2) are readily utilized and removed by plants.

To further extend the actual understanding of the factors influencing Se foraging and accumulation in plant species, future studies should focus on investigating the role of both biotic and abiotic factors in the uptake and accumulation of Se, such as exploring the plant-microbe interactions and the impact of pH, soil type, and irrigation practices.

## Data Availability

All data generated during the current study and additional pictures are available from the corresponding author upon reasonable request.
